# LncRNA MALAT1/miR‐129 axis promotes glioma tumorigenesis by targeting SOX2

**DOI:** 10.1111/jcmm.13667

**Published:** 2018-05-29

**Authors:** Zhiyong Xiong, Luyang Wang, Qiangping Wang, Ye Yuan

**Affiliations:** ^1^ Department of Neurosurgery Union Hospital Tongji Medical College Huazhong University of Science and Technology Wuhan China; ^2^ Department of Neurology The Central Hospital of Wuhan Tongji Medical College Huazhong University of Science and Technology Wuhan China

**Keywords:** glioma, lncRNA MALAT1, miR‐129, proliferation, *SOX2*

## Abstract

We aimed to explore the interaction among lncRNA MALAT1, miR‐129 and SOX2. Besides, we would investigate the effect of MALAT1 on the proliferation of glioma stem cells and glioma tumorigenesis. Differentially expressed lncRNAs in glioma cells and glioma stem cells were screened out with microarray analysis. The targeting relationship between miR‐129 and MALAT1 or *SOX2* was validated by dual‐luciferase reporter assay. The expressions of MALAT1, miR‐129 and *SOX2* mRNA in both glioma non‐stem cells and glioma stem cells were examined by qRT‐PCR assay. The impact of MALAT1 and miR‐129 on glioma stem cell proliferation was observed by CCK‐8 assay, EdU assay and sphere formation assay. The protein expression of SOX2 was determined by western blot. The effects of MALAT1 and miR‐129 on glioma tumour growth were further confirmed using xenograft mouse model. The mRNA expression of MALAT1 was significantly up‐regulated in glioma stem cells compared with non‐stem cells, while miR‐129 was significantly down‐regulated in glioma stem cells. MALAT1 knockdown inhibited glioma stem cell proliferation via miR‐129 enhancement. Meanwhile, miR‐129 directly targeted at *SOX2* and suppressed cell viability and proliferation of glioma stem cells by suppressing *SOX2* expression. The down‐regulation of MALAT1 and miR‐129 overexpression both suppressed glioma tumour growth via SOX2 expression promotion in vivo. MALAT1 enhanced glioma stem cell viability and proliferation abilities and promoted glioma tumorigenesis through suppressing miR‐129 and facilitating *SOX2* expressions.

## 1 INTRODUCTION

Glioma is one of the most prevalent and aggressive malignant tumours in human central nervous system with high mortality rate around the world.^1^ Nowadays, although many advances have been achieved in treatment strategies, such as immunotherapy, stereotactic radiotherapy and new chemotherapy drugs, the recurrence rate and mortality rate were still not reduced because of metastasis.^2^, ^3^ Moreover, due to our poor understanding of glioma pathogenesis, therapeutic strategies for glioma are limited.^4^ Glioma stem cells (GSCs) are a subgroup of glioma cells with the ability of self‐renewal,^5^, ^6^ and are remarkably resistant to chemotherapy and radiotherapy in malignant gliomas.^7^ Thus, it is imperative to focus on the mechanisms of glioma tumorigenesis by elucidating GSCs.

Long non‐coding RNAs (lncRNAs), a group of non‐protein coding transcripts longer than 200 nucleotides, play a key role in many types of malignant tumours, including glioma.^8^ LncRNAs may function as competing endogenous RNAs (ceRNAs) to modulate miRNA expressions.^9^ Metastasis‐associated lung adenocarcinoma transcript 1 (MALAT1) is an lncRNA with a length of approximately 8000‐nt.^8^ Wang et al revealed that MALAT1 was aberrantly expressed in carcinoma cells.^10^ Ma et al^11^ found that MALAT1 was up‐regulated in glioma tissues and correlated with the progression of glioma. However, the molecular mechanism of MALAT1 in glioma cells remains unknown.

MicroRNAs (miRNAs) are short single‐stranded RNA molecules which function as a critical modulator in gene expression by wholly or partially binding to corresponding mRNAs.^12^ MiRNAs play a crucial role in the progression of various cancers, such as osteosarcoma 12 and glioma.^13^ MiR‐129 is a miRNA family mainly composed of miR‐129‐3p, miR‐129‐2‐3p and miR‐129‐5p.^14^ Previous researches have already demonstrated that miR‐129‐5p acted as a tumour inhibitor in multiple cancers such as ovarian cancer,^15^ breast cancer 16 and also glioma.^17^ Chen et al^18^ found that Notch‐1/ E2F7/Beclin‐1 axis was regulated and further the viability of malignant glioma cells could be impaired by up‐regulation of miR‐129. However, the role of miR‐129 in glioma stem cells has not been elucidated so far.

SRY (sex determining region Y)‐box 2 (*SOX2*) is revealed as a stemness marker in glioma 19 and a transcription factor highly associated with pluripotency.^20^ Current study investigated the relationship between *SOX2* and miR‐129 to further elucidate the molecule network of miR‐129 in glioma stem cells progression. Activated SOX2 expression maintained stemness and self‐renewal of GSCs.^21^ SOX2 is associated with GBM stem‐like phenotype which is more resistant to γ‐radiation.^22^ Therefore, SOX2 expression changes are significant for GSC stemness maintainment.

This study hypothesized the promoter role of lnc MALAT1 in glioma and validated its effect in both in vivo and in vitro experiments. MALAT1 was suspected to bind to miR‐129 which target at SOX2, an oncogene, and their interaction was depicted in this study. By illustrating the underlying mechanism that facilitated glioma progression, this study may contribute to the application of glioma target therapy.

## 2 MATERIALS AND METHODS

### 2.1 Clinical specimens

Fourteen histologically verified glioma tissue specimens based on the WHO‐2007 classification from 2015‐2017 were obtained from patients treated with surgery at the Union Hospital, Tongji Medical College, Huazhong University of Science and Technology. Inclusion criteria were the following: (*i*) WHO graded gliomas confirmed by histopathology. Exclusion criteria were as follows: (*i*) an unknown IDH1‐mutation status, (*ii*) patients with a previous history of brain tumours, (*iii*) patients younger than 18 years of age. All glioma tissues were first preserved in liquid nitrogen and stored at −80°C for the subsequent experiment. This project was ratified by the Ethics Committee of the Union Hospital, Tongji Medical College, Huazhong University of Science and Technology and written informed consents were collected from all patients.

### 2.2 Glioma cell culture and stem cell isolation

Glioma cells were cultured in Dulbecco's modified Eagle medium (DMEM)/high glucose with 10% fetal bovine serum (FBS, Gibco, Carlsbad, CA, USA) and were maintained in a humidified incubator at 37°C with 5% CO_2_. GSCs were separated as described previously from tissue cells.^23^, ^24^ Primary cells were detached with trypsin, washed once in FACs buffer (PBS containing 1%‐2% BSA and 5 mM EDTA), then stained with anti‐CD24‐FITC (Invitrogen, Carlsbad, CA, USA) and anti‐CD133‐PE (Invitrogen) using 10 μl of antibody per 10^6^ cells, and incubated at 4°C for 15 minutes. Following incubation, cells were washed once with FACs buffer. The CD44^+^CD24^−^ cells were considered to be GSCs.

### 2.3 Microarray analysis

GSE23806 microarray data obtained from Gene Expression Omnibus database was applied to the filtration of aberrantly expressed lncRNAs. “Match matrix” file from GEO website (https://www.ncbi.nlm.nih.gov/geo/query/acc.cgi?acc=GSE23806) were downloaded and processed for samples and corresponding probes. Corresponding platform information of GPL570 ([HG‐U133_Plus_2] Affymetrix Human Genome U133 Plus 2.0 Array) was downloaded and processed as well for matching probes and corresponding gene symbols. Expressions of lncRNAs in 14 conventional glioma cell lines (G121, G84, G120, G61, G112, G130, U118MG, SF268, G124, G118, G142, G44, SW1783 and G22) and 5 glioma stem‐like cell lines (GS01, GS02, GS03, GS04 and GS05) were analysed using unpaired T‐test method in the LIMMA package and the obtained *P* values were adjusted with Benjamini‐Hochberg method. A volcano plot filtering (fold change > 4, adjusted *P*‐value < .01) was drawn and all the differentially expressed lncRNAs were listed in the heat map.

### 2.4 QRT‐PCR

The extraction of total RNA was implemented by Trizol reagent (Invitrogen, USA), and the purity and concentration of RNA were detected by NanoDrop 1000 (Nanodrop Technologies Inc., USA). First Strand cDNA Synthesis Kit (Biochemical Industry Inc., USA) was used for the reverse transcription according to the instructions. Quantitative PCR was conducted through Bio‐Rad MiniOpticon real‐time PCR system, using Maxima SYBR Green qPCR Master Mix (2X) kit (Fermentas, Burlington, Canada) for amplified detection according to the manual. GAPDH was deemed as a reference gene of *SOX2* and MALAT1, while U6 was an internal control of miR‐129. The relative expression levels of MALAT1, miR‐129 and *SOX2* were determined by using the 2^−ΔΔCT^ method. The primers were synthesized by Sangon Biotech (Shanghai, China). The detailed primers were exhibited in Table 1.

**Table 1:** Primer sequences of qRT‐PCR

	Primer sequence (5′‐3′)	
MALAT1	Sense	ATGCGAGTTGTTCTCCGTCT
	Anti‐Sense	TATCTGCGGTTTCCTCAAGC
miR‐129	Sense	CGGCGGTTTTTTGCGGTCTGGGCT
	Anti‐Sense	CAACCTGGAGGACTCCATGCTG
SOX2	Sense	GGAGTTGTCAAGGCAGAGAAG
	Anti‐Sense	CGCCGCCGATGATTGTTAT
GAPDH	Sense	ACCCCGCCGCCTGTGGArGG
	Anti‐Sense	TTCTGACGGCAGGTCAGGT
U6	Sense	TCGAACAGGAGGAGCAGAGAGCGA
	Anti‐Sense	TCGAACAGGAGGAGCAGAGAGCGA

### 2.5 Western blot

The isolation of total protein from tissues and protein concentration measurement were performed by using RIPA lysis buffer and BCA protein concentration kit (Beyotime, Shanghai, China). Proteins were segregated by SDS‐PAGE and then transferred onto PVDF membranes (Invitrogen) in accordance with the instructions, followed by blocking in 5% nonfat milk for 60 minutes and incubation with primary antibodies against *SOX2* (ab137385, 1:1000, Abcam corporation) at 4°C overnight. After that, the membranes were washed with Tris Buffered Saline Tween (TBST) every 5 minutes for four times and then incubated in HRP‐conjugated goat anti‐rabbit IgG secondary antibody (1:2000) for 2 hours. After being rinsed twice in TBST, the immunoreactive bands were developed using an enhanced chemiluminescence detection system (Amersham Pharmacia Biotech, Buckinghamshire, England).

### 2.6 Cell transfection

Glioma stem cells in logarithmic growth were first seeded in the culture dish, and then placed onto the 6‐well plate when cell growth reached 80%‐90% confluence. MALAT1 siRNA, miR‐129 mimics and miR‐129 inhibitor were synthesized by Genepharma Company (Shanghai, China). 293T cells were respectively transfected with lncRNA MALAT1 siRNA, miR‐129 mimics, miR‐129 inhibitor and negative control lncRNA MALAT1 or negative control mimics using Lipofectamine^™^ 2000 reagent (Life Technologies Inc., USA) following the instructions. After transfection for 48 hours, the sample was collected and transfection efficiency was detected. The experimental groups were generally divided into five groups as follows: Blank group (without transfection), Negative Control (NC) group (transfected with negative control lncRNA MALAT1 or mimics), si‐MALAT1 group (transfected with lncRNA MALAT1 siRNA), miR‐129 group (transfected with miR‐129 mimics) and miR‐129 inhibitor group (transfected with miR‐129 inhibitor).

### 2.7 Dual‐luciferase reporter gene assay

Plasmid pmirGLO vectors were bought from Promega Corporation (Madison, USA). The recombinant reporter gene pmir‐GLO‐MALAT1 and pmir‐GLO‐*SOX2* was constructed. Glioma stem cells were seeded into the 24‐well culture plate until 90% confluency. Then Recombinant vector (wild‐type or mutated type) was transfected into the cells together with miR‐129 mimics or mimics control by using Lipofectamine 2000 reagent, followed by incubation for 48 hours. After that, the fluorescence intensity of transfected cells was examined by luciferase reporter assay kit (Promega, Madison, WI, USA).

### 2.8 CCK‐8 assay

Cell proliferation was assessed by Cell Counting Kit‐8 (CCK‐8; Beyotime, Shanghai, China). Transfected glioma stem cells were seeded into 96‐well culture plate at a density of 2 × 10^3^ cells per well containing 10 μL CCK‐8 solutions and cultured overnight. The optical density (OD) value of each well was assessed at 24, 48, 72 and 96 hours using a microplate reader. Absorbance was recorded at 450 nm. The assay was repeated three times.

### 2.9 EdU assay

Transfected glioma stem cells were cultured in 96‐well plates. Briefly, glioma stem cells were incubated with EdU labelling medium at moderate concentration for 2 hours. The cells were then fixed with 0.5% TritonX‐100 in PBS (100 μL) for 25 minutes, and stained with 100 μL Apollo dye solution (Ribobio) for 30 minutes at room temperature. The cells were subsequently stained using DAPI (Invitrogen) and incubated for half an hour. The percentage of EdU positive cells was calculated using ImageJ software.

### 2.10 Sphere formation assay

Glioma stem cells were seeded into 6‐well plate at 5 × 10^3^ cells/mL in the culture medium containing 20 ng/mL basic Fibroblast Growth Factor (bFGF) and Epidermal Growth Factor (EGF), 5 L g/mL insulin (Sigma‐Aldrich,St. Louis, MO, USA), 0.4% BSA (Invitrogen, USA) medium and 0.02% B27 (Invitrogen). After incubation for 7 days, cells were fixed using 10% formalin and photographed under a conventional microscope. Sphere Formation Efficiency (SFE) representing the ability of sphere formation (diameter > 75 μm) was calculated. The formula of SFE was: the numbers of cell sphere in each well / the total number of cells originally seeded in each well.

### 2.11 Xenograft mouse model

Nine male nude mice (5 weeks old) were purchased from Shanghai Experimental Animal Centre (Shanghai, China). Animal experiments were strictly conducted in accordance with the protocols of Animal Ethics Committee of Union Hospital, Tongji Medical College, Huazhong University of Science and Technology. MALAT1 shRNA sequence was sub‐cloned into the pGLV2‐U6‐Puro vector (Genepharma), the lentivirus was packaged in 293T cells according to the protocol and then infected glioma cells at the multiplicity of infection (MOI) of 20. Stable MALAT1 knockout cells were selected using 1 μg/mL puromycin. AgomiR‐129 (Rio‐bio, Guangzhou, China) was then transfected into glioma stem cells using Lipofectamine 2000 (Invitrogen). After transfection, cells were collected and washed thrice with ice‐cold PBS and then suspended in PBS. 2 × 10^6^ transfected glioma stem cells were injected into the left flanks of nude mice. The mice were sacrificed executed 28 days after injection. Mice injected with glioma stem cells without transfection was diagnosed as control group. Tumour volumes were detected every week. Tumour volume was monitored by measuring the length and width and calculated using the formula: *V* = (*L* × *W* ^2^) × 0.5. At 28th day after implantation, mice were sacrificed and isolated tumours were weighed. In addition, total proteins were extracted from the tumour samples and detected by Western blot.

### 2.12 Statistical analysis

Statistical analyses were performed through GraphPad Prism 6.0 (GraphPad Software, San Diego, California, USA). All quantitative data are presented as means ± standard deviation (SD). Differences between groups were compared using Student's *t*‐test while comparison in multiple groups was practiced with one‐way ANOVA. Benjamini‐Hochberg was used to adjust multiple testing for selection of differentially expressed lncRNAs. Statistical significance was based on *P* value < .05.

## 3 RESULTS

### 3.1 MALAT1 is highly expressed in glioma stem cells

**Figure 1:**
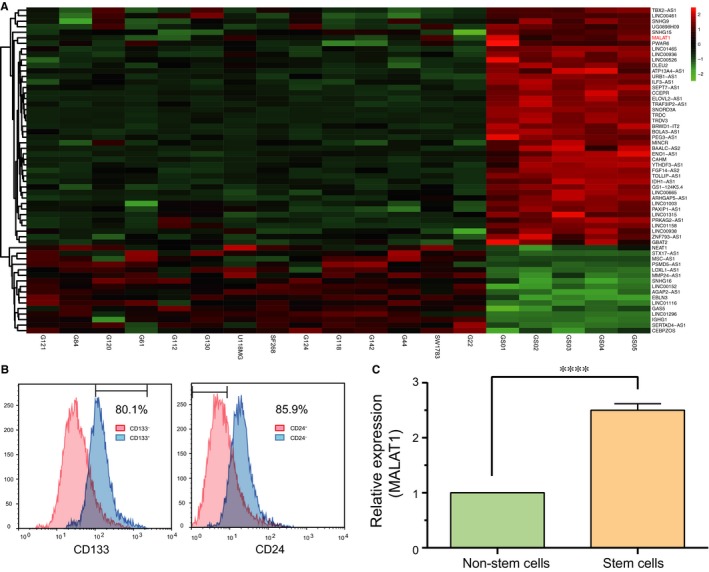
MALAT1 is highly expressed in glioma stem cells. A, The differentially expressed lncRNAs in glioma stem cells (including GS01, GS02, GS03, GS04 and GS05) and glioma tissue cells (including G121, G84, G120, G61, G112, G130, U118MG, SF268, G124, G118, G142, G44, SW1783 and G22) were screened through microarray analysis. MALAT1 was found to be significantly up‐regulated in GSCs. B, Glioma stem cells were isolated from glioma cancer cells with flow cytometry and those with CD144^+^ CD24^−^ markers were considered as GSCs. C, The expression level of MALAT1 in glioma stem cells was remarkably higher than that in glioma tissue cells examined by qRT‐PCR. ****P <* *.0001, compared with cancer cells (glioma tissue cells)

The differentially expressed lncRNAs, including 43 up‐regulated lncRNAs and 16 down‐regulated lncRNAs, were screened out in glioblastoma stem‐like cells and conventional glioma cells based on the filtration criteria of fold change > 4 and adjusted *P *< .01 (Table 2, Figure 1A). Microarray analysis results revealed that MALAT1 was significantly up‐regulated in glioma stem‐like cells. To validate the prediction, we used 14 patient tissue samples to isolate GBCs. Those CD133^+^CD24^−^ cells were regarded as GSCs as reported previously.^25^, ^26^ Characteristics of patients were listed in Table 3. After we obtained glioma stem cells from glioma cells, qRT‐PCR was utilized to measure the expression level of MALAT1. As shown in Figure 1B,C, MALAT1 expression in glioma stem cells was remarkably higher than compared with non‐stem cells (*P *< .0001).

**Table 2:** The results of fold‐change and *P* value of differential genes

Gene symbol	logFC	*P*.Value	Adj. *P*.Value
TRDV3	8.575920353	1.69E‐18	1.36E‐15
TRDC	8.182903102	8.45E‐21	1.02E‐17
SNORD3A	7.003737202	2.01E‐23	4.85E‐20
LINC01158	6.679955378	1.45E‐07	2.99E‐06
LINC00461	4.680745748	0.001802104	0.007229372
LINC01003	4.616437767	4.15E‐08	1.13E‐06
FGF14‐AS2	4.250067783	2.40E‐11	2.52E‐09
CAHM	4.035562004	2.63E‐13	5.77E‐11
FGF14‐AS2	3.778449578	4.34E‐10	2.56E‐08
LINC00936	3.764411621	1.24E‐09	6.25E‐08
PEG3‐AS1	3.719100724	3.95E‐13	7.95E‐11
ARHGAP5‐AS1	3.53093737	9.48E‐10	4.98E‐08
PAXIP1‐AS1	3.412324551	3.62E‐07	6.43E‐06
CCEPR	3.056910193	1.11E‐15	5.34E‐13
ILF3‐AS1	3.054249994	1.97E‐11	2.38E‐09
ZNF793‐AS1	2.966212306	1.21E‐08	4.29E‐07
YTHDF3‐AS1	2.876594005	4.92E‐11	4.40E‐09
GS1‐124K5.4	2.859079983	5.22E‐11	4.51E‐09
MINCR	2.856789138	1.16E‐07	2.58E‐06
UG0898H09	2.840202417	0.000173758	0.001067752
URB1‐AS1	2.724625797	3.22E‐12	4.58E‐10
LINC01315	2.591253926	1.04E‐08	3.86E‐07
TRAF3IP2‐AS1	2.546802754	4.06E‐15	1.64E‐12
BAALC‐AS2	2.54080024	3.42E‐11	3.31E‐09
MALAT1	2.523271109	3.14E‐05	0.000261728
IDH1‐AS1	2.491701012	2.70E‐12	4.35E‐10
DLEU2	2.488769448	1.57E‐09	7.57E‐08
TOLLIP‐AS1	2.479583102	3.21E‐17	1.94E‐14
LINC00665	2.464888348	2.14E‐11	2.43E‐09
BOLA3‐AS1	2.457662529	4.87E‐14	1.47E‐11
PWAR6	2.443116861	7.73E‐05	0.000546506
SEPT7‐AS1	2.422505772	1.00E‐13	2.69E‐11
ATP13A4‐AS1	2.395337515	1.58E‐10	1.09E‐08
PRKAG2‐AS1	2.367825007	1.32E‐07	2.85E‐06
TBX2‐AS1	2.316519621	0.000103617	0.000697036
ELOVL2‐AS1	2.243879181	7.32E‐15	2.52E‐12
GBAT2	2.216352939	1.43E‐06	2.00E‐05
ENO1‐AS1	2.175228792	1.62E‐10	1.09E‐08
LINC01465	2.161541462	1.28E‐10	9.40E‐09
LINC00526	2.158895263	4.93E‐10	2.77E‐08
MALAT1	2.135200437	0.000172118	0.001061614
SNHG9	2.087679222	9.08E‐05	0.000621076
LINC00938	2.029574413	2.45E‐06	3.19E‐05
SNHG15	2.00342226	0.000455262	0.002349268
BRWD1‐IT2	2.000447174	1.33E‐13	3.21E‐11
CEBPZOS	−2.030429459	4.17E‐05	0.000332317
MMP24‐AS1	−2.08914189	1.63E‐05	0.000152843
DLEU2	−2.098699898	0.000268268	0.001504038
EBLN3	−2.114406503	1.09E‐08	4.00E‐07
SNHG16	−2.286897084	2.15E‐07	4.05E‐06
NEAT1	−2.33227962	0.002655131	0.009759727
GAS5	−2.431689548	2.76E‐07	5.06E‐06
PSMD5‐AS1	−2.451857035	0.000929539	0.004203816
AGAP2‐AS1	−2.673571961	1.16E‐07	2.58E‐06
SERTAD4‐AS1	−2.758723401	1.42E‐05	0.000137251
LINC00152	−3.068202392	2.36E‐09	1.09E‐07
STX17‐AS1	−3.299781124	0.000265796	0.001503275
MSC‐AS1	−3.711235086	0.002113499	0.008140511
IGHG1	−3.984993575	2.70E‐06	3.47E‐05
LINC01116	−4.329934135	8.08E‐07	1.25E‐05
LOXL1‐AS1	−5.600952763	7.29E‐06	7.79E‐05
LINC01296	−5.95432695	1.69E‐06	2.33E‐05

Negative values (−) denote low expression and positive denote high expression. LogFC represents the gene expression fold change of stem cells compared with cancer cells. *P*.Value and adj. *P*.Value refer to statistical values, *P *<* *.01 indicates statistical significance.

**Table 3:** Patient characteristics

Factor	Number
Patients	14
Gender (m, f)	10/4
Age, y (median, range)	47.6 (18.1‐84.0)
Surgical procedure	
Biopsy	238
Surgery	62
WHO grade	
II	2
III	6
IV	6
Tumour location	
Frontal	3
Temporal	6
Parietal	2
Occipital	1
Midline/basal ganglia/corpus callosum	2
IDH1 R132H status	
Wild‐type	8
Mutated	6

### 3.2 MALAT1 promotes the growth of glioma stem cells

**Figure 2:**
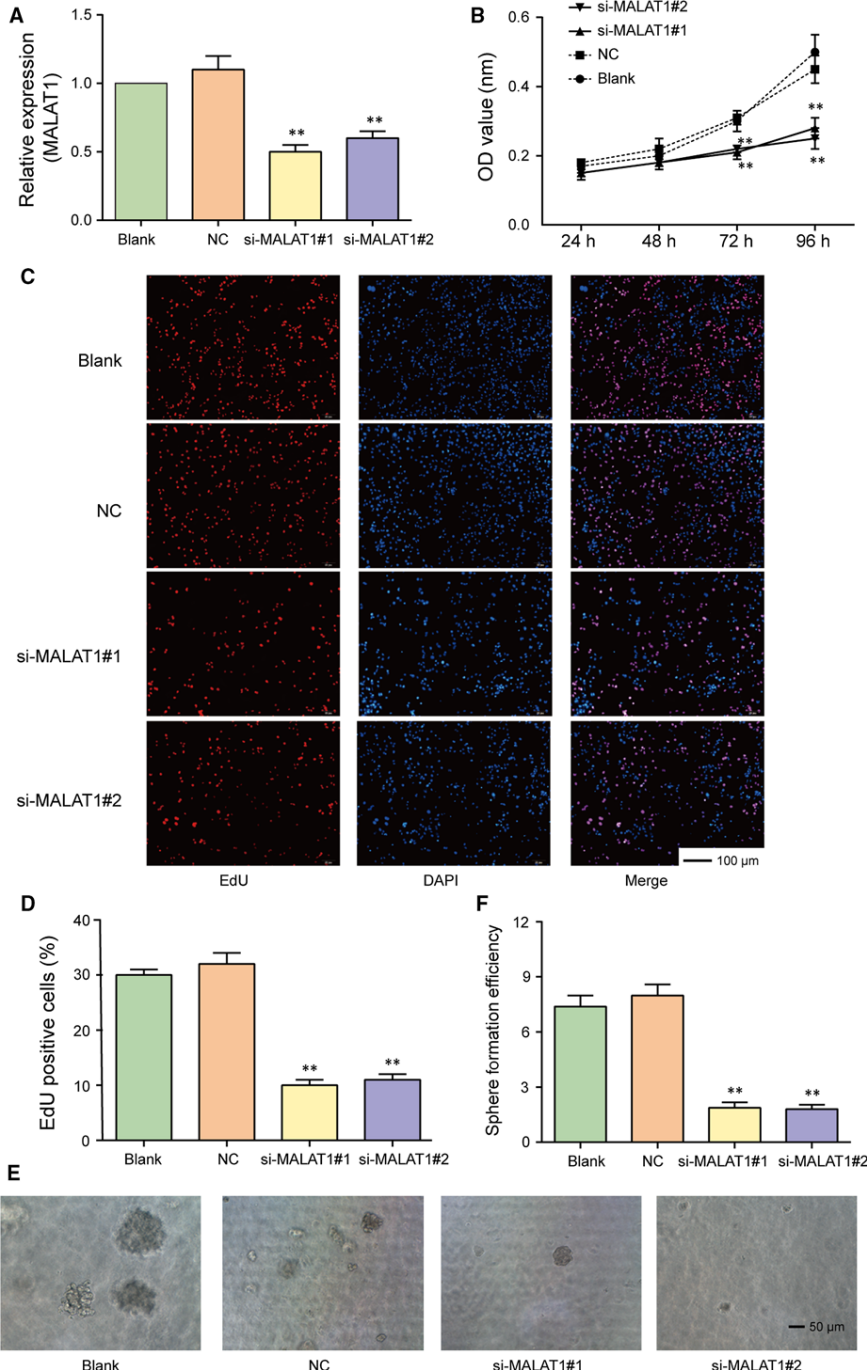
MALAT1 promotes the growth of glioma stem cells. A, The expression of MALAT1 in glioma stem cells significantly decreased after transfection with si‐MALAT1 (si‐MALAT1#1 or si‐MALAT1#2). B, The cell growth of glioma stem cells transfected with si‐MALAT1 was suppressed significantly confirmed by CCK‐8 assay. C‐D, The percentage of EdU positive cells transfected with si‐MALAT1 dramatically declined, suggesting that knockdown of MALAT1 could significantly repress the proliferation of glioma stem cells. E‐F, The sphere formation efficiency of glioma stem cells in si‐MALAT1 groups significantly decreased in comparison with NC group detected by sphere formation assay. Scale bar, 50 μm. **P <* *.01, compared with NC group

After transfection with si‐MALAT1 (si‐MALAT1#1 or si‐MALAT1#2), the expression of MALAT1 in glioma stem cells significantly decreased (Figure 2A, *P* < .01). The result of CCK‐8 assay suggested that cell viability in two si‐MALAT1 groups significantly reduced in comparison with NC group, indicating that cell growth of GBCs was significantly suppressed by si‐MALAT1 (Figure 2B, both *P *< .01). Meanwhile, the percentage of EdU positive cells in si‐MALAT1 group also dramatically declined, suggesting that knockdown of MALAT1 could significantly repress the proliferation of glioma stem cells (Figure 2C,D, both *P *<* *.01). In addition, sphere formation assay revealed that the sphere formation efficiency in si‐MALAT1 groups significantly decreased in comparison with NC group (Figure 2E,F, both *P *<* *.01). Therefore, we concluded that MALAT1 promoted GSCs proliferation.

### 3.3 MiR‐129 is a potential target of MALAT1

**Figure 3:**
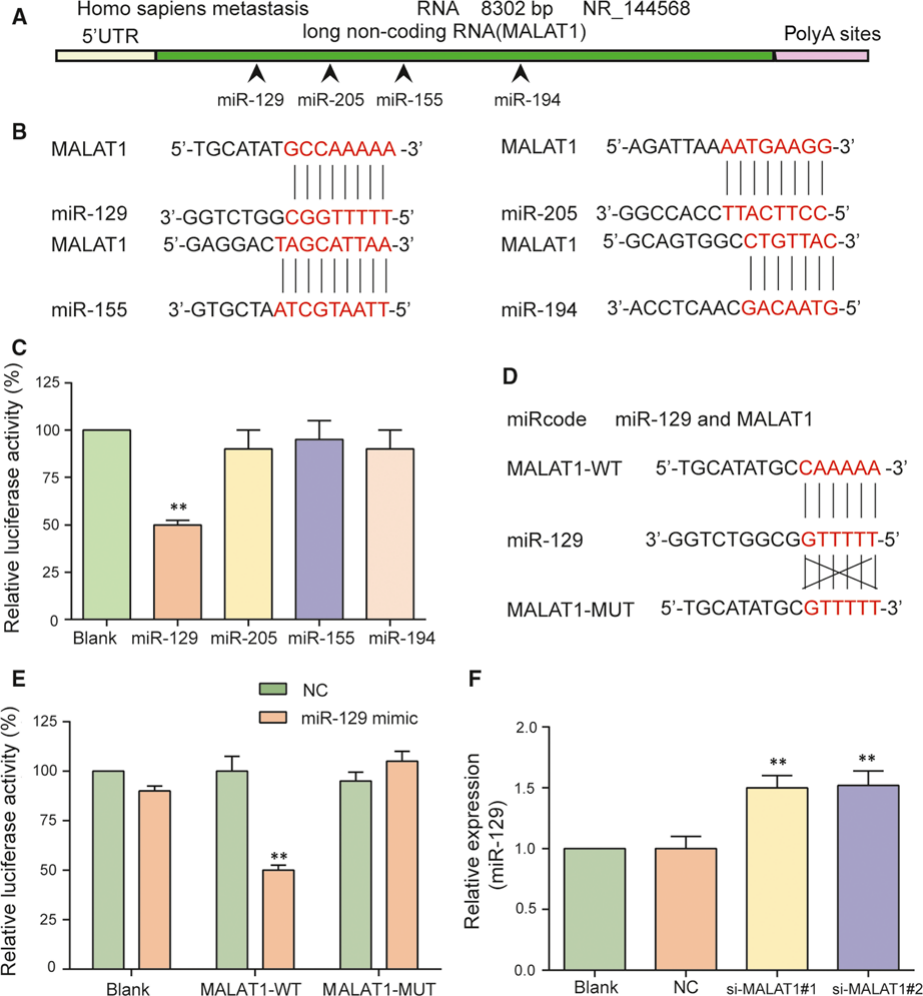
MiR‐129 is a potential target of MALAT1. A‐B, The binding sites of MALAT1 and miR‐129, miR‐205, miR‐155 as well as miR‐194 were predicted by miRcode. C, Compared with blank group, the luciferase activity in miR‐129 group significantly decreased, while there was no significant difference between miR‐205, miR‐155, miR‐194 and blank group. D‐E, Verified by dual luciferase reporter gene assay, MALAT1 could directly target miR‐129. F, The expression level of miR‐129 was significantly up‐regulated in si‐MALAT1#1 and si‐MALAT1#2 groups in comparison with NC group examined by qRT‐PCR. **P <* *.01, compared with blank group

Predicted by miRcode database (http://www.mircode.org/), potential targeting relationships between MALAT1 and miR‐129, miR‐205, miR‐155, miR‐194, as well as the binding sites were presented in Figure 3A,B. Dual‐luciferase reporter assay results indicated that only miR‐129 mimics restrained the luciferase activity of the reporting vector containing MALAT1 sequence significantly (Figure 3C, *P* < .01). We also constructed a MALAT1‐WT 3′ UTR and a MALAT1‐MUT 3′ UTR luciferase reporter vectors to validate the direct targeting relationship between miR‐129 and MALAT1. The luciferase activity of the cells co‐transfected with MALAT1‐WT 3′ UTR and miR‐129 mimics group was considerably weaker (Figure 3D,E, *P* < .01). Additionally, we also examined the expression of miR‐129 using qRT‐PCR after si‐MALAT1 transfection. As shown in Figure 3F, the expression level of miR‐129 was significantly up‐regulated in si‐MALAT1#1 and si‐MALAT1#2 groups in comparison with NC group (both *P *<* *.01).

### 3.4 MiR‐129 restrains the propagation of glioma stem cells

**Figure 4:**
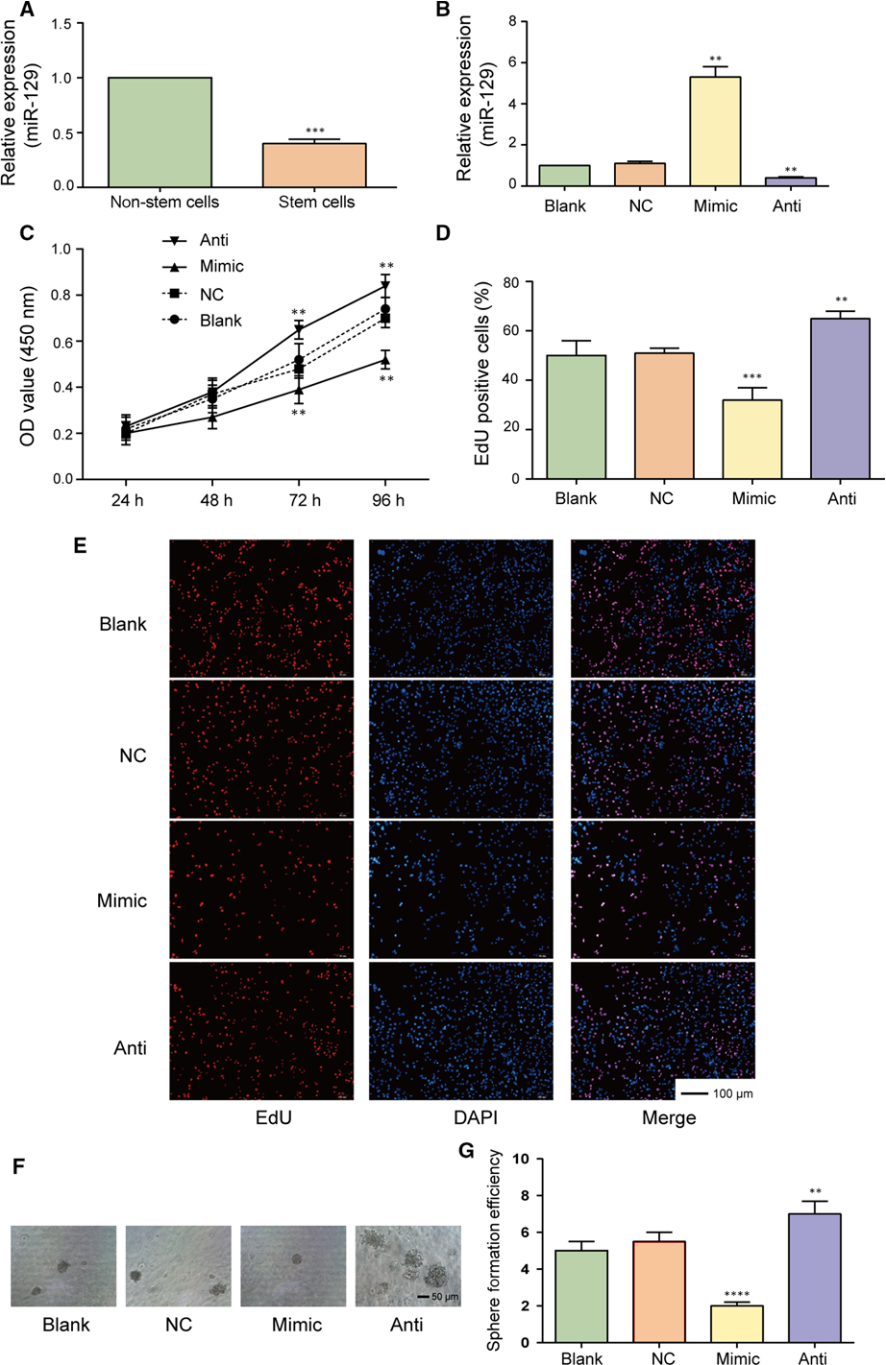
MiR‐129 suppresses the proliferation of glioma stem cells. A, The expression of miR‐129 in glioma stem cells was significantly lower than that in the glioma tissue cells detected by qRT‐PCR. B, The expression of miR‐129 in miR‐129 mimics group dramatically increased, while that in miR‐129 inhibitor group significantly decreased observed by qRT‐PCR. C‐E, CCK‐8 assay and EdU assay showed that cell proliferation of glioma stem cells were significantly attenuated in miR‐129 overexpression group but remarkably enhanced in miR‐129 inhibitor group compared with NC group. F‐G, The sphere formation assay results also revealed that the sphere formation efficiency of glioma stem cells in miR‐129 mimics group was significantly lower than that in NC group, while the cells transfected with miR‐129 inhibitor presented higher sphere formation efficiency in comparison with NC group determined by sphere formation assay. Scale bar, 100 μm. **P <* *.01. ***P <* *.001, ****P <* *.0001, compared with NC group

The results of qRT‐PCR indicated that miR‐129 expression in glioma stem cells was remarkably lower than that in non‐stem cells (Figure 4A, *P* < .001). Moreover, after being transfected with miR‐129 mimics, the expression of miR‐129 in glioma stem cells dramatically increased, while the expression level of miR‐129 in cells transfected with miR‐129 inhibitor significantly decreased (Figure 4B, both *P *<* *.01). The results from CCK‐8 assay and EdU assay suggested that the glioma stem cell viability was significantly attenuated in miR‐129 overexpression group, whereas the proliferation ability of glioma stem cells in miR‐129 inhibitor group was drastically enhanced compared with NC group (Figure 4C‐E, all *P *<* *.01). Meanwhile, the results of sphere formation assay indicated that the sphere formation efficiency of glioma stem cells in miR‐129 mimics group was notably lower (*P *<* *.0001), while the cells transfected with miR‐129 inhibitor presented a higher sphere formation efficiency in comparison with NC group (Figure 4F,G, *P* < .01). Therefore, overexpression of miR‐129 could significantly suppress the proliferation ability and stemness of glioma stem cells.

### 3.5 *SOX2* is a direct target of miR‐129

**Figure 5:**
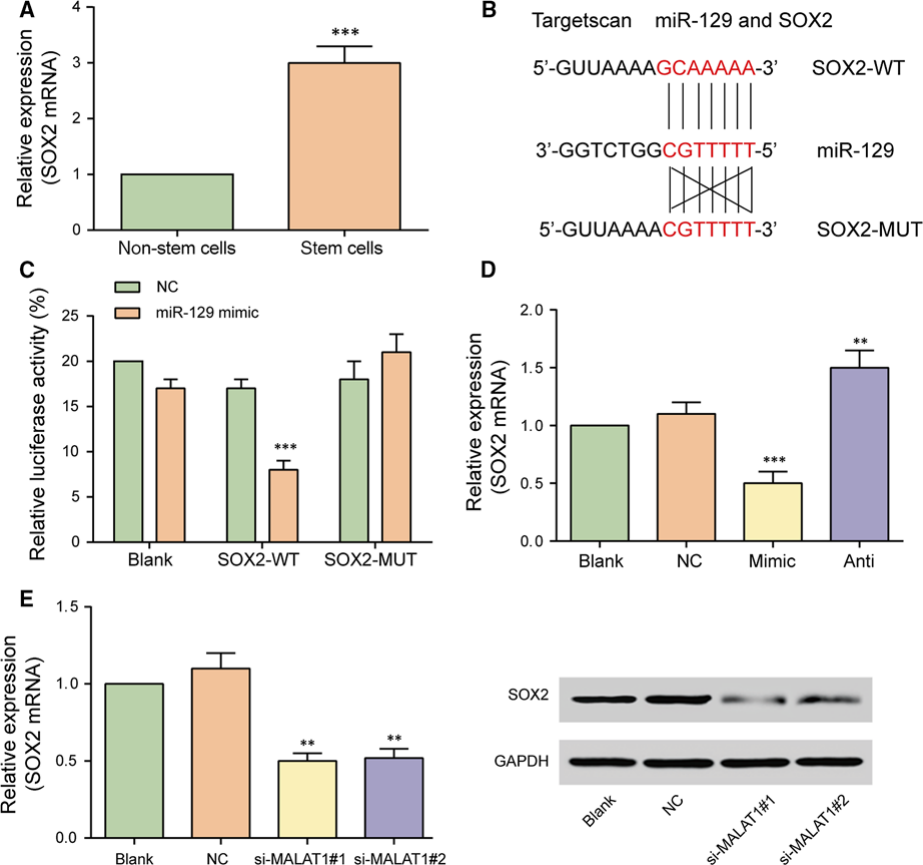
*SOX2* is a direct target of miR‐129. A, The expression of *SOX2* mRNA in glioma stem cells was significantly higher than that in glioma tissue cells detected by qRT‐PCR. ***P <* *.001, compared with cancer cells (glioma tissue cells). B, *SOX2* was a candidate target of miR‐129 predicted by TargetScan. C, The targeted relationship between miR‐129 and *SOX2* was validated by dual luciferase reporter gene assay. D, The expression of *SOX2* in glioma stem cells transfected with miR‐129 mimics remarkably decreased, while glioma stem cells transfected with miR‐129 inhibitor presented a significant increase in the expression of *SOX2*. E, Compared with NC group, *SOX2* mRNA and protein expression drastically decreased after transfected with si‐MALAT1#1 and si‐MALAT1#2 detected by qRT‐PCR and Western blot respectively. **P <* *.01, ***P <* *.001, all compared with NC group

Previous studies ever identified that *SOX2* was required for cancer cell line progression in lung and esophageal squamous carcinoma, highlighting the importance of *SOX2* as a lineage‐survival oncogene.^27^ High *SOX2* expression has been associated with several human solid tumours, including glioma.^28^ The present study evaluated that expression of *SOX2* mRNA in glioma stem cells was significantly higher than that in glioma cells detected by qRT‐PCR (Figure 5A, *P* < .001). TargetScan predicted the direct targeting relationship between miR‐129 and SOX2 and the dual‐luciferase reporter assay validated their direct targeting relationship since the luciferase activity in *SOX2*‐WT + miR‐129 mimics group was remarkably weaker compared with that in NC group (Figure 5B,C, *P* < .001). Hence, *SOX2* was a direct target of miR‐129. Furthermore, miR‐129 mimic suppressed *SOX2* expression, while miR‐129 inhibitor enhanced *SOX2* expression (Figure 5D, *P* < .01). In addition, after being transfected with MALAT1 siRNA (si‐MALAT1#1 or si‐MALAT1#2), the mRNA and protein expressions of SOX2 were significantly down‐regulated (Figure 5E, *P* < .01). We thus confirmed negative regulation between miR‐129 and *SOX2* and positive regulation between MALAT1 and *SOX2*.

### 3.6 The down‐regulation of MALAT1 expression and miR‐129 overexpression suppresses glioma tumour growth in vivo

**Figure 6:**
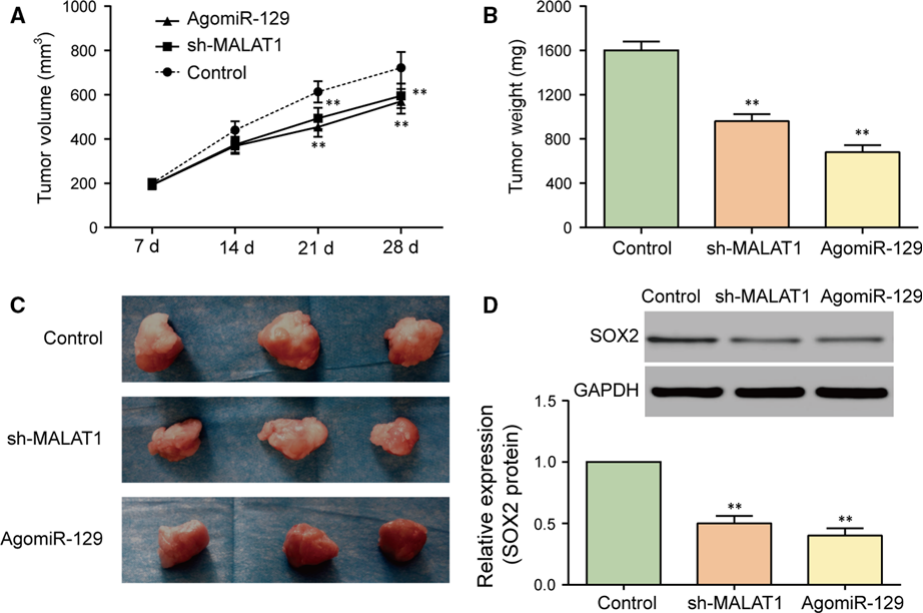
Either MALAT1 down‐regulation or miR‐129 overexpression suppresses glioma tumour growth in vivo. A‐C, The tumour volume and weight of mice transfected with sh‐MALAT1 and AgomiR‐129 significantly reduced compared with control group. D, The expression of SOX2 protein in mice transfected with sh‐MALAT1 or AgomiR‐129 was significantly down‐regulated. **P <* *.01, compared with control group

Xenograft mice models were established by subcutaneously injecting glioma stem cells with different expression of MALAT1 and miR‐129. The tumour size was measured every 7 days. At the 28th day, mice were sacrificed and tumours were obtained and weighed. The results disclosed that the tumour volume and weight in sh‐MALAT1 and AgomiR‐129 groups significantly reduced in comparison with control group (Figure 6A‐C, all *P *<* *.01). Meanwhile, western blot was used to examine the relative protein expression of SOX2 in mice. Consistent with the previous experiment results, the expression of SOX2 protein in mice tumour was significantly down‐regulated after stable transfection with sh‐MALAT1 or AgomiR‐129 mimics (Figure 6D). Overall, down‐regulation of MALAT1 or overexpression of miR‐129 suppressed glioma tumour growth in vivo.

## 4 DISCUSSION

Glioma has been regarded as a kind of aggressive malignancy of the central nervous system worldwide. Glioma stem cells play essential role in glioma tumorigenesis. Previous studies have revealed that MALAT1 functions as oncogenic lncRNA in multiple cancers.^29^ Chen et al^30^ reported that the expression of MALAT1 was down‐regulated in preeclampsia, suppressing proliferation and cell cycle of JEG‐3 cells. Ma et al^11^ revealed that MALAT1 was correlated with the malignant status in glioma. Li et al^9^ found that MALAT1 expression was up‐regulated in glioma, promoting cell propagation and inhibiting cell apoptosis. Consistent with previous research results, our data demonstrated that MALAT1 was involved in glioma tumorigenesis as a tumour promoter. Through CCK‐8 proliferation assay, EdU assay and sphere formation assay, we also found that MALAT1 could promote the proliferation of glioma stem cells.

Emerging evidence has indicated that lncRNA could sponge miRNA and regulate the functions of miRNAs.^31^ Previous studies also indicated that MALAT1 presented various functions as miRNA sponges in different cancers. For example, MALAT1 promoted malignant melanoma growth and metastasis by sponging miR‐22.^32^ In our research, MALAT1 bound to miR‐129 directly to suppress its expression, which was firstly studied.

Earlier studies have reported that miR‐129‐2 suppressed glioma by targeting *HMGB1* in glioma in an DNA methylation way.^33^ MiR‐129 also impair human malignant glioma progression via autophagic flux enhancement by regulating a novel Notch‐1/ E2F7/Beclin‐1 axis.^18^ Kang et al^34^ revealed that miR‐129‐2 suppressed proliferation of esophageal carcinoma cells through down‐regulation of SOX4 expression. Elevated SOX2 enforces glioblastoma stem cell identity.^35^ High miR‐21/low SOX2 axis was capable of classifying patients with longer survival.^36^ We also diagnosed miR‐129 as a tumour suppressor via SOX2 modulation firstly in glioma tumorigenesis. Besides, the MALAT1/miR‐129/SOX4 was of great significance in present molecular system investigation.

As an important pluripotent marker of stem cells, *SOX2* has been recognized as serving a crucial role in maintaining the properties of cancer stem cells.^37^ According to the study of Wang et al^38^ *SOX2* was considered as a predictor of survival in gastric cancer to inhibit cell proliferation and metastasis. Chen et al also revealed that silencing of *SOX2* could regulate the apoptosis rate of human lung cancer cells.^39^ In our research, we speculated that the interaction between miR‐129 and *SOX2* was associated with the suppression of glioma tumorigenesis and the inhibition of glioma stem cell activities. The results of experiments indicated that miR‐129 inhibited the expression of *SOX2*, suppressing GSCs proliferation. On the contrary, MALAT1 promoted the expression of *SOX2*, thus boosting the viability and proliferation of GSCs. Additionally, the effects of MALAT1 and miR‐129 on the glioma tumour were confirmed in a xenograft mouse model, indicating that MALAT1 promoted glioma tumour growth by regulating miR‐129 and *SOX2*. However, some concerns still existed in the current study. For example, MALAT1 could bind to other miRNAs in glioma stem cells. The downstream pathway of *SOX2* MALAT1/miR‐129/SOX4 axis could further been investigated as well.

## 5 CONCLUSION

In summary, our research showed that MALAT1 was up‐regulated in glioma stem cells and acted as a tumour promoter in glioma progression. Silencing of MALAT1 suppressed the proliferation and stemness of GSCs and in vivo tumour growth via up‐regulating miR‐129 and further inhibiting *SOX2*, providing a promising therapy target for the treatment of glioma.

## CONFLICT OF INTEREST

The authors confirm that there are no conflicts of interest.

## AUTHOR CONTRIBUTION

Zhiyong Xiong and Luyang Wang contributed to research conception and design as well as manuscript drafting; Ye Yuan analysed and interpreted data; Luyang Wang made statistical analysis; Qiangping Wang revised the manuscript and took the role of funding collectors. In addition, all authors approved final manuscript.
